# Correction: Gold nanoparticles grafted with chemically incompatible ligands

**DOI:** 10.1039/d1ra90112e

**Published:** 2021-05-05

**Authors:** Joanna M. Wolska, Aleksandra Błażejewska, Martyna Tupikowska, Damian Pociecha, Ewa Górecka

**Affiliations:** Faculty of Chemistry, University of Warsaw Pasteura 1 02-093 Warsaw Poland jokos@chem.uw.edu.pl +48 22 822 0211

## Abstract

Correction for ‘Gold nanoparticles grafted with chemically incompatible ligands’ by Joanna M. Wolska *et al.*, *RSC Adv.*, 2021, **11**, 9568–9571, DOI: 10.1039/D1RA00547B.

The authors regret that one of the affiliations (affiliation *a*) was incorrectly shown in the original manuscript. The corrected list of affiliations is as shown below.

The Royal Society of Chemistry apologises for these errors and any consequent inconvenience to authors and readers.

## Supplementary Material

